# Axonal and dendritic density field estimation from incomplete single-slice neuronal reconstructions

**DOI:** 10.3389/fnana.2014.00054

**Published:** 2014-06-25

**Authors:** Jaap van Pelt, Arjen van Ooyen, Harry B. M. Uylings

**Affiliations:** ^1^Computational Neuroscience Group, Department of Integrative Neurophysiology, Center for Neurogenomics and Cognitive Research, VU University AmsterdamAmsterdam, Netherlands; ^2^Department of Anatomy and Neuroscience, VU University Medical CenterAmsterdam, Netherlands

**Keywords:** neuronal morphology, reconstruction, slices, density fields, cut branches, recovery

## Abstract

Neuronal information processing in cortical networks critically depends on the organization of synaptic connectivity. Synaptic connections can form when axons and dendrites come in close proximity of each other. The spatial innervation of neuronal arborizations can be described by their axonal and dendritic density fields. Recently we showed that potential locations of synapses between neurons can be estimated from their overlapping axonal and dendritic density fields. However, deriving density fields from single-slice neuronal reconstructions is hampered by incompleteness because of cut branches. Here, we describe a method for recovering the lost axonal and dendritic mass. This so-called completion method is based on an estimation of the mass inside the slice and an extrapolation to the space outside the slice, assuming axial symmetry in the mass distribution. We validated the method using a set of neurons generated with our NETMORPH simulator. The model-generated neurons were artificially sliced and subsequently recovered by the completion method. Depending on slice thickness and arbor extent, branches that have lost their outside parents (orphan branches) may occur inside the slice. Not connected anymore to the contiguous structure of the sliced neuron, orphan branches result in an underestimation of neurite mass. For 300 μm thick slices, however, the validation showed a full recovery of dendritic and an almost full recovery of axonal mass. The completion method was applied to three experimental data sets of reconstructed rat cortical L2/3 pyramidal neurons. The results showed that in 300 μm thick slices intracortical axons lost about 50% and dendrites about 16% of their mass. The completion method can be applied to single-slice reconstructions as long as axial symmetry can be assumed in the mass distribution. This opens up the possibility of using incomplete neuronal reconstructions from open-access data bases to determine population mean mass density fields.

## Introduction

Cognition emerges from electrical activity dynamics in neuronal networks in the brain. These networks consist of a large number of neurons from a multitude of cell types connected to each other via synapses. Neurons innervate space through their axonal and dendritic arborizations and synapses may be formed when axonal and dendritic arbors are sufficiently close in space (Peters, 1979; Mishchenko et al., 2010). The shape of neuronal arborizations is therefore a crucial determinant of synaptic connectivity in the brain. Neurons show a large variability in shape but maintain characteristics typical for their cell type. The distribution of dendritic and axonal mass in space can be described as a mass density field, indicating at each location in space the amount of dendritic and axonal mass (in terms of length or volume). Averaging these density fields over a population of neurons gives a statistical representation of how this cell type distributes its mass in space.

**Figure 1:**
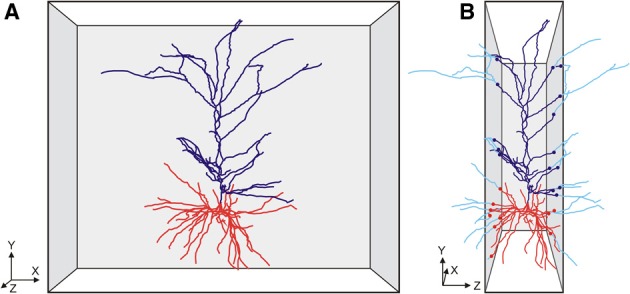
**Cartoon of (A) a front view of a slice with a pyramidal neuron extending its apical (dark blue) and basal (red) dendritic branches in the XY direction of the slice, and (B) a side view of the slice with the neuron having many branches cut in the Z-direction of the slice**. The cut endings are indicated by colored dots with the lost branches indicated in light blue. Axonal branches are not shown in these cartoon images.

Potential locations for synaptic connections between neurons can be found by searching all spatial locations where axonal and dendritic branches are sufficiently close to each other (Van Pelt et al., 2010). In a recent study we showed that the number of potential locations can also be derived from the overlap between axonal and dendritic density fields (Van Pelt and Van Ooyen, 2013). Thus, for creating neuronal networks with neurons at different locations in space and originating from a variety of cell types, knowledge about their population mean density fields is sufficient to estimate the number of potential synapse locations between these neurons. Note, however, that for estimating actual connectivity additional knowledge is required of the probability that a synapse will develop at a potential location (e.g., Mishchenko et al., 2010). Thanks to open-access data bases neuronal reconstructions from a large number of cell types have now become widely available [e.g., NeuroMorpho.org (Ascoli, 2006) and SenseLab (Shepherd et al., 1997)]. These data could, in principle, be used for calculating population mean density fields for each cell type represented in the data base. However, many of the reconstructions originate from stained neurons in single slices with thicknesses up to about 300 μm. With axonal and dendritic arborizations extending beyond the spatial boundaries of single slices, all these reconstructions have cut endings and are thus incomplete (Figure 1). This incompleteness hampers the use of these reconstructions for density field estimations.

In the present study a method is developed to estimate the lost axonal and dendritic parts by extrapolating densities calculated from the observed parts inside the slices to the space outside the slices, assuming axial symmetry in the axonal and dendritic mass distribution. This so called completion procedure is described in the section Materials and Methods and applied to three different data sets of rat cortical layer 2/3 pyramidal neurons for which axial symmetry can be assumed. A vital part of the study is the validation of the method. To this end, 50 neurons generated with our NETMORPH simulator (Koene et al., 2009) were artificially sliced with varying slice thicknesses, and subsequently subjected to the completion procedure. Comparison of the original mass distributions with the sliced ones and the completed ones showed the level of recovery obtained.

For slices of thickness of 300 μm or thicker the validation study showed that the completion procedure resulted in a (almost) full recovery of the dendritic and axonal mass density fields from the incomplete reconstructed neurons. An important aspect in the recovery of cut arborizations is the occurrence of orphan branches, i.e., branches which have lost their parents located outside the slice. These orphan branches are no longer part of the contiguous reconstructed structure and thus result in an underestimate of the mass of the arborization inside the slice, which cannot be recovered by the completion method. This especially occurred for larger arborizations (axons) in thin slices of 100 or 200 μm thickness.

## Materials and methods

### Mass densities in 3D space

**Figure 2:**
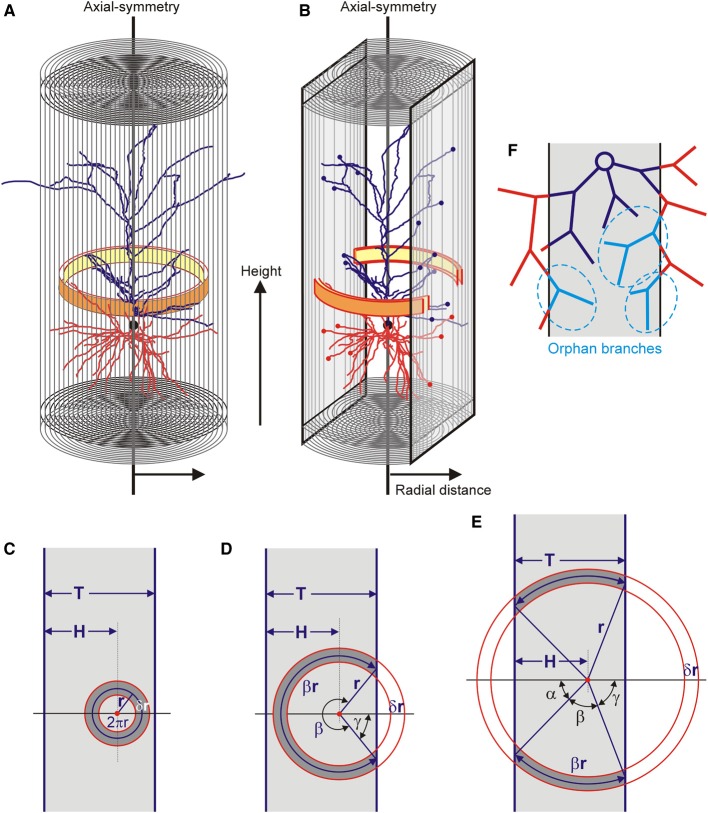
**(A)** An axial radial coordinate system is used and axial symmetry is assumed. The distribution of mass is obtained by summing all the mass per integration ring (yellow-orange ring) at a given axial position (height) and with a given radius. The final distribution is given as a function of radius and height. **(B)** Within the restricted space of a slice, the integration ring becomes fractionated when it exceeds the slice boundary. **(C–E)** Three different conditions for the integration ring in a slice of thickness *T*. The integration ring has its center at a distance *H* from one of the boundaries, has a radius *r* (pointing to the center of the ring) and a thickness δ*r*. It can **(C)** be fully contained in the slice, **(D)** extend the slice on one of its sides, or **(E,B)** extend on both of its sides. **(F)** Effect of slicing of a neuron leaving a contiguous structure within the slice (dark blue), lost branches outside the slice (red) and orphan branches within the slice (light blue and marked in dashed ellipses). These orphan branches have lost their parent branches and therefore their connection to the dark blue contiguous structure.

The completion method is based on the calculation of the neuronal mass densities inside the slices and extrapolating these densities to the area outside the slices. For this extrapolation it is assumed that the mass densities of the neuronal arborizations are axial symmetric (Figure 2A). This is a plausible assumption for pyramidal neurons, which have the apical main shaft as symmetry axis. Using an axial-radial coordinate system, the neuronal mass is determined for a given height and a given radius, thus summing all the mass in a ring as is illustrated in Figure 2A. For a fully intact neuron, the radius of the integration ring ranges from zero up to the maximal radial extension of the arborizations. The total mass distribution of a neuron becomes a function of height and radius. Evidently, in the case of pyramidal neurons, these distributions can also be obtained separately for axons and (apical and basal) dendrites. For a sliced neuron, its radial extension in the XY direction of the slice can become as large as in the intact neuron, but in the Z-direction it is limited by the boundary planes of the slice. Also the integration ring will be complete for small radii but becomes incomplete at one or two sides for larger radii. In the last case the ring is reduced to two separate ring segments (Figure 2B). The estimation of the mass densities is then restricted to the remaining parts of the integration rings.

### Method for estimating mass densities outside the slices

With the assumption of axial symmetry, the densities in the fractional parts of the integration rings inside the slice can be extrapolated to their complementary parts outside the slice. The volume of the integration ring inside and outside the slice is fully determined by the position of its center (i.e., the symmetry axis), the radius of the integration ring, and the thickness of the slice. Three situations can be distinguished. The ring can be fully contained in the slice, it can extend the slice on one side, or it can extend the slice on both sides (Figures 2C–E). The neuronal masses can only be obtained for the volume fraction of the ring inside the slice. For an integration ring with radius *r* (pointing to the center of the ring), the volume fraction *F*(*r*) of the ring area (or volume) within the slice can be expressed in terms of the thickness of the slice (*T*), the position of the ring center within the slice (*H*) and the radius of the ring (*r*).

- Ring fully within slice:
  *H* > *r* and *T* − *H* > *r*.
  Ring volume fraction: *F*(*r*) = $\frac{2\pi r\delta r}{2\pi r\delta r}$ = 1.
- Ring extends the slice on one of its sides:
  *H* > *r* and *T* − *H* ≤ *r*, with γ = *arccos* ($\frac{T-H}{r}$).
  Ring volume fraction: *F*(*r*) = $\frac{\beta r\delta r}{2\pi r\delta r}$ = $\frac{\beta }{2\pi }$ = $\frac{2\pi -2\gamma }{2\pi }$ = $\frac{\pi -arccos\left(\frac{T-H}{r}\right)}{\pi }$.
- Ring extends on both sides of the slice:
  *H* ≤ *r* and *T* − *H* ≤ *r*, with α = *arccos* ($\frac{H}{r}$) and γ = *arccos* ($\frac{T-H}{r}$).
  Ring volume fraction: *F*(*r*) = $\frac{2\beta r\delta r}{2\pi r\delta r}$ = $\frac{\beta }{\pi }$ = $\frac{\pi -\alpha -\gamma }{\pi }$ = $\frac{\pi -arccos\left(\frac{H}{r}\right)-arccos\left(\frac{T-H}{r}\right)}{\pi }$.

An estimate of the mass in a full ring *M*_*ring*_(*r*, *h*, δ*r*, δ*h*) with radius *r* and at height *h* and with radial thickness δ*r* and height δ*h* can be obtained by dividing the experimentally observed neuronal mass *M*_*obs*_(*r*, *h*, δ*r*, δ*h*) in the part of the ring inside the slice by the ring volume fraction. Thus,

$$M_{ring}(r,\;h,\;\delta r,\;\delta h)\;\approx \;\frac{M_{obs}(r,\;h,\;\delta r,\;\delta h)}{F(r)}.$$

For the mass density in the ring *D*_*ring*_ (*r*, *h*) we now obtain

$$D_{ring}\text{​}\left(r,\;h\right)=\frac{M_{ring}(r,\;h,\;\delta r,\;\delta h)}{V_{ring}}=\frac{M_{ring}(r,\;h,\;\delta r,\;\delta h)}{\text{2}\pi r\delta r\delta h}$$

with *V*_*ring*_ the volume of the full ring. In our analysis, integration rings with a radial thickness of δ*r* = 1 μm and height δ*h* = 1 μm are used.

### Orphan branches

Neuronal arborizations may extend beyond the boundaries of a slice but more distal parts may bend back into the slice (Figure 2F). Such branches have lost their parent branches and are called orphan branches. Orphan branches are no longer connected with the proximal parts of the arborization inside the slice. As the reconstruction procedure quantifies only the contiguous parts of the arborization inside the slice, the orphan branches are lost. In the calculation of the mass densities from the contiguous part inside the slice, this then results in an underestimation of the neuronal mass. As the completion procedure extrapolates from this neuronal mass, it does not solve the problem of lost orphan branches.

### Slice thickness and position of neurons inside a slice

For the completion procedure it is important to know the thickness of the slice and the position of the cell body with respect to both boundary planes of the slice (Z-axis). Although the thickness of the slices is provided in most reconstructions, this is not the case for the cell’s position in the slice, so that we had to estimate the cell’s position from the reconstructions themselves. For this estimation we used the fact that all cut terminal tips have approximately the same Z-coordinate. To this end we calculated the Z-coordinates of all terminal tips of both axons and dendrites in the reconstruction and obtained a frequency distribution along the Z-axis. When the distribution showed a sudden increase in frequency (number of tips) at one or both of its ends, this was interpreted as the effect of cutting. When a frequency increase was observed at the low-Z end of the distribution, the cut part of the cell’s arborization was aligned at the low-Z end of the slice. When a frequency increase was observed at the high-Z end of the distribution, the cut part of the cell’s arborization was aligned at the high-Z end of the slice. Finally, when a frequency increase was observed at both sides of the distribution, it indicated the actual thickness of the slice. The thickness information and the estimated cell positions were used in the completion procedure.

### Population averages of the mass densities

The mass density completion procedure was applied to the arborizations of individual neurons because the procedure depends on the position of the individual neuron inside the slice, which varies from neuron to neuron. To obtain a population average of the estimated mass densities, the cells were aligned by their somata and all the cells were rotated in such a way that their apical main stem was pointing into the Y-direction.

### Alignment procedure

For the alignment of the apical dendrite, the orientation of the apical main stem was needed. As the data formats in the reconstruction files generally do not distinguish between apical main stem, and apical tuft and oblique branches, this information needed to be derived from the supplied data. To this end, an iterative procedure was applied of pruning terminal line pieces off the apical dendrite, until it was reduced to a single segment with one terminal line piece. The tip coordinate of this terminal line piece together with the exit coordinate from the soma of the apical dendrite provided the alignment line piece with which to rotate the whole cell in the XY plane (i.e., around the Z-axis) in such a way that the projection of the alignment line piece onto the XY plane was pointing into the Y-direction. Alternatively, a rotation in the XYZ space could have been applied so that the alignment line pieces were all pointing into the Y-direction. However, such a rotation would also have changed the Z-coordinates of the terminal tips in the arborizations and would have hampered the use of tip coordinates for estimating the cell’s position in the slice. As a consequence, the orientation of the alignment segment could maintain a slightly tilted angle with respect to the Z-axis, while the Z-axis itself was taken as symmetry axis for the calculation of the mass distribution. Given the wide spread of arborization mass, the effect of a possibly slightly tilted orientation on the final estimated mass distribution was assumed to be negligible.

### Validation

Crucial for the completion procedure is its validation, i.e., whether the masses of the completed neurons are equal to those of the original non-sliced neurons. Such a validation is not possible for experimental data sets of sliced neurons, but can be done for a set of model-generated neurons. For this validation, we used a set of 50 neurons, generated with our NETMORPH simulator (Koene et al., 2009). These model neurons were subsequently sliced according to several slice thicknesses, followed by the density field completion procedure. The masses of the original and of the completed neurons were subsequently compared. Because in this case the mass of orphan branches was also known, the comparisons were made from completions with and without inclusion of orphan branches.

### Data sets

#### NETMORPH-generated neurons

The data set of neuronal arborizations used for validating the mass completion procedure was obtained with our simulator NETMORPH (Koene et al., 2009). A number of 50 random neuron morphologies were generated with growth parameters optimized on a set of rat cortical L2/3 pyramidal neurons, reconstructed by Svoboda (Shepherd and Svoboda, 2005) and made available by the NeuroMorpho.org data base (Ascoli, 2006). This same data set was also used and described in an earlier study (Van Pelt and Van Ooyen, 2013).

#### Svoboda data set

This dataset consists of 11 young adults (25–36 days PN) Sprague Dawley rat somatosensory barrel cortex L2/3 pyramidal neurons, reconstructed by Svoboda (Shepherd and Svoboda, 2005) from 300 μm thick slices, and made available by the NeuroMorpho.org database (Ascoli, 2006).

#### Markram data set

This dataset consists of 33 young (13–15 days PN) Wistar rat somatosensory cortex L2/3 pyramidal neurons, reconstructed by Wang et al. (2002) from 300 μm thick slices, and made available by the NeuroMorpho.org database (Ascoli, 2006).

#### Parnavelas-Uylings data set

This dataset originates from a study on basal dendritic development in female Sprague-Dawley rat visual cortex pyramidal and non-pyramidal neurons (Parnavelas and Uylings, 1980; Uylings et al., 1994). Reconstructions of 153 Golgi stained pyramidal dendrites from slices with a thickness of about 120 μm were obtained from layer 2/3 at different ages of postnatal cortical development (10, 14, 18, 24, 30, and 90 days PN).

## Results

### Validation—data NETMORPH

**Figure 3:**
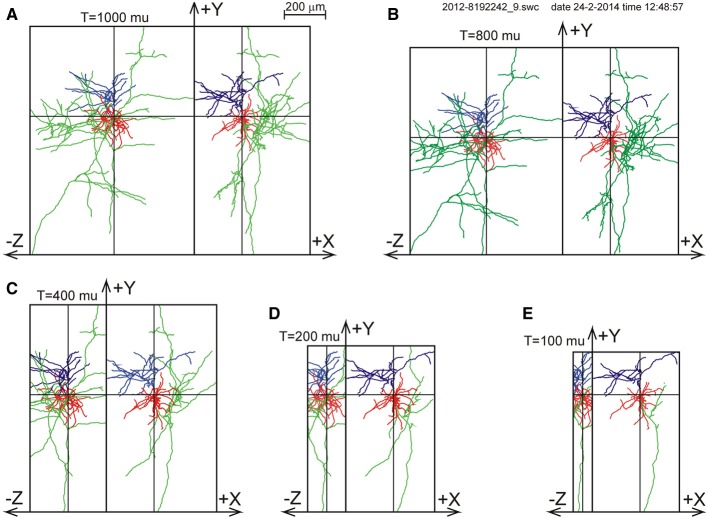
**Illustration of slicing of a NETMORPH neuron for slice thicknesses *T* of (A) 1000 μm, (B) 800 μm, (C) 400 μm, (D) 200 μm, and (E) 100 μm**. The neurons are plotted as projections onto the XY plane and onto the YZ plane. Neurons have their somata in the center of the slice and are aligned with their apical dendrite pointing into the Y-direction. The Z-axis denotes the depth of the slice. Note that slicing in the Z-direction also influences the neuronal ranges in the X- and Y-directions. The panels show the axonal arborizations in green, the basal dendrites in red and the apical dendrites in blue.

For one of the NETMORPH-generated neurons an example is given in Figure 3 to demonstrate the impact of slicing on the remaining morphology within the slice.

To validate the completion procedure, the 50 NETMORPH generated neurons were artificially sliced with slice thicknesses of 100, 200, 300, 400, 500, 600, 1000, or 2000 μm. First, the neurons were aligned with their apical main stem pointing into the Y-direction, and with the slicing perpendicular to the Z-axis. For the position of the neurons in the slice, two choices could be made. With the somata positioned in the center of the XY plane of the slice, the Z-coordinate was put either in the center of the slice (thus with equal distances to both cutting sides of the slice), or was uniform randomly selected within an 80% Z-range (between 10 and 90%) or within a 60% Z-range (between 20 and 80% of the Z-range). After slicing, the axial-radial mass distribution was obtained for each individual neuron followed by the completion procedure, which was applied excluding or including orphan branches. The results of the completion procedure were finally summed and averaged over all the NETMORPH neurons in the data set.

**Figure 4:**
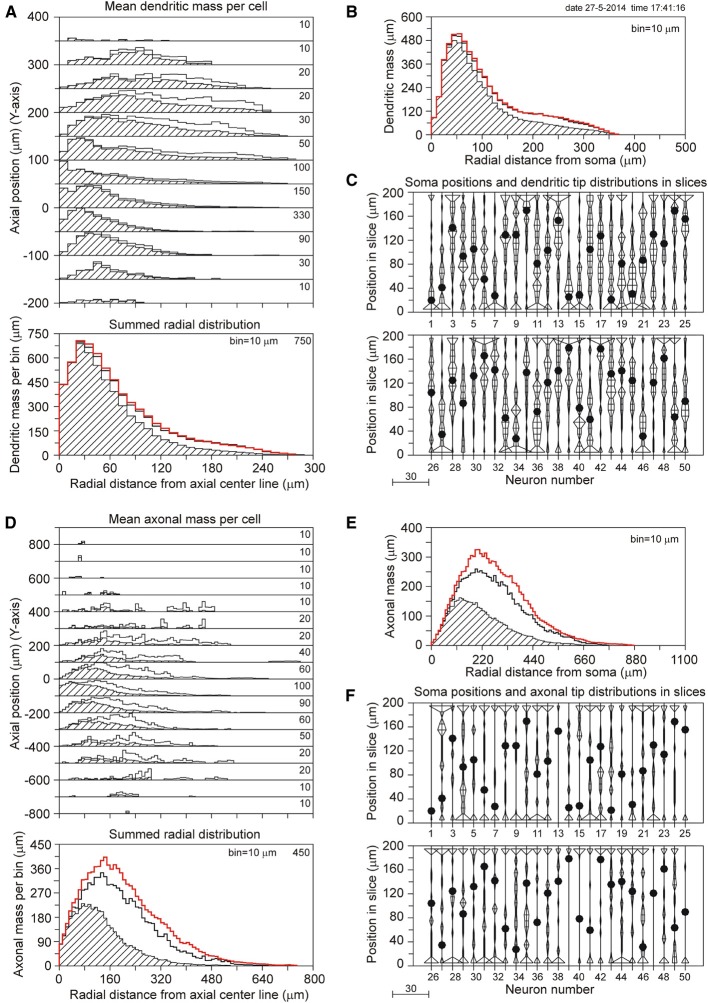
**Completion of sliced NETMORPH neurons with a slice thickness of 200 μm**. Orphan branches were not included in the completion procedure. **(A)** Axial-radial distribution of mean dendritic mass per neuron. The dashed distributions show the mass as obtained within the slice, while the solid black curves show the mass distributions after completion. The axial axis (Y-axis) is binned with bins of 50 μm, containing their respective radial distributions, which are scaled according to the numbers in the upper right corner in each bin. Thus the mass distribution at a Y-position of -50 μm has a maximum mass of 330 μm per radial bin of 10 μm. The bottom graph in **(A)** shows the radial dendritic mass distribution summed over all axial Y-positions. The black curve for the completed mass distribution has a strong overlap with the red curve for the full non-sliced dendrites. **(B)** Distribution of dendritic mass as a function of the radial distance to the soma (this distribution is similar to a 3D Sholl diagram). Again, there is strong overlap between the black curve for the completed mass distribution and the red curve for the full non-sliced dendrites. **(C)** Graph illustrating the positions of the cell bodies (solid circles) of the 50 neurons along the depth of the slice (Z-axis). In this example the cell positions were uniform randomly chosen in an 80% range of the full depth. The graph also includes the frequency distributions of the dendritic tip Z-coordinates per neuron. With a binning of 10 μm each frequency per bin is plotted as a horizontal line piece centered in the bin and symmetrical around the vertical position axis. The tips of these line pieces are subsequently connected to each other. The frequency scale is indicated by the small bar left underneath the panel. Clearly is shown how the number of tips (length of the horizontal line piece) can accumulate at the boundary planes, indicating the presence of cut endings. Panels **(D–F)** show similar results for the axonal arborizations.

**Figure 5:**
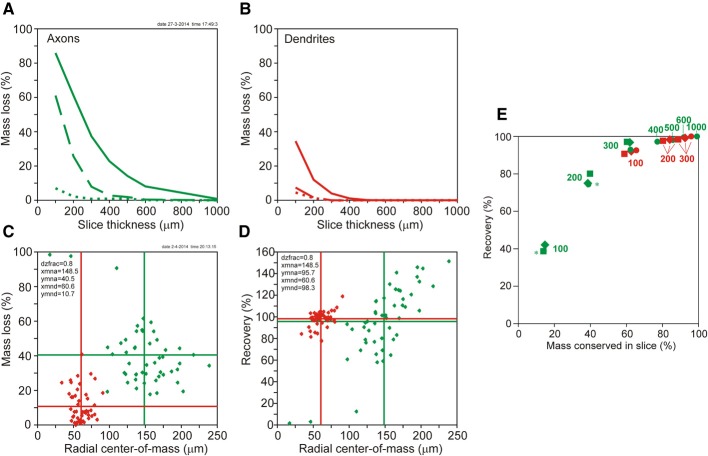
**(A,B)** Plots of mass loss (%) vs. slice thickness of NETMORPH generated neurons centrally located in the artificial slice. (**A**, solid green curve) Loss of axonal mass by slicing. (**A**, dashed green curve) Remaining axonal mass loss mass after completion without orphan branches. (**A**, dotted green curve) Remaining axonal mass loss after completion including orphan branches. (**B**, solid red curve) Loss of dendritic mass by slicing of NETMORPH generated neurons centrally located in the slice. (**B**, dashed red curve) Remaining dendritic mass loss after completion without orphan branches. (**B**, dotted red curve) Remaining dendritic mass loss after completion including orphan branches. **(C,D)** Plots of mass loss by slicing and recovery by completion vs. the mean radial center-of-mass of the sliced axonal and dendritic arborizations. The 50 NETMORPH generated neurons were artificially sliced by 300 μm thick slices. The neuronal somata were uniform randomly located within a 80% range of the slice thickness. The original 50 NETMORPH generated neurons had a mean dendritic radial center of mass at 70 μm and a mean axonal radial center of mass at 204 μm. **(C)** Scatterplot of the mass loss of the individual sliced axonal (green) and dendritic (red) arborizations. **(D)** Scatterplot of the recovery by completion of the individual sliced axonal (green) and dendritic (red) arborizations. **(E)** Plot of recovery values vs. the mass conserved in the slices. The data points (excluding orphan branches) are labeled with the slice thickness (in μm). The positioning of the somata in the slices is indicated by circles for the central position, by squares for the 80% range and by drops for the 60% range random positioning. Axonal data are plotted in green and dendritic data in red. Overlap of data points is indicated by a star.

The results for a slice thickness of 200 μm, 80% range random Z-coordinates of the somata, and ignoring orphan branches are shown in Figure 4. Figure 4A displays the axial-radial mean dendritic mass distribution, in the top part for several axial positions (summed per 50 μm height steps) and in the bottom part summed over all heights. The dashed histograms present the masses of the sliced dendrites, while the solid lines present the completed dendritic mass distributions. Both distributions coincide at small radial distances from the axis, but the completed ones become increasingly higher at larger radial distances. Evidently this is caused by the larger correction factors at higher radial distances. The distribution for the non-sliced original full dendrites is also included in the bottom panel of Figure 4A as a red solid line. The total mass of 5264 μm of the dashed (sliced) distribution deviated 19.7% from the original dendritic mass of 6552 μm, while the completion procedure resulted in a mass of 6399 μm, deviating only 2.3% from the original mass (Table 1). Figure 4B displays the mean mass distribution as a function of the radial distance to the soma of the sliced dendrites (dashed), of the completed ones (black solid line), and of the original non-sliced dendrites (red solid line). This distribution is related to a 3D Sholl diagram which counts the number of intersections with a set of concentric spheres (in practice, however, the Sholl method is most frequently applied to 2D projections, see Uylings and Van Pelt (2002) for a discussion on Sholl diagrams). The tail in both distributions originates from the apical dendrites. Figure 4C shows the positions of the somata in the Z-direction of the slice (filled circles) as well as the distribution of Z-coordinates of the terminal tips per neuron. The effect of cutting is clearly seen in the accumulation of terminal tips at the boundary layers of the slice. Figures 4D–F show the findings for the mean axonal mass distribution. Note that panel 4D extends over larger axial (Y-axis) and radial distances than panel 4A for the dendritic mass distribution. The summed radial distribution shows a substantial difference between the sliced axonal distribution (dashed) and the completed one (black solid line), with a mean total axonal mass per neuron of 4239 μm for the sliced axon and of 8531 μm for the completed mass, while the original axon (red solid line) had a total length of 10655 μm. Thus, in 200 μm thick slices, 60.2% of the original axonal mass was lost, while the completion procedure reduced the axonal loss to 19.9% (Table 1). Comparison of the black and red solid lines in the bottom panel of Figure 4D clearly shows the level of axonal recovery obtained in the case of 200 μm thick slices. The difference in the level of recovery between dendrites and axons appears to be related to the fraction of conserved mass in the slice (see the results shown in Figure 5E).

**Table 1:** **Effect of slicing on the length of apical and basal dendrites and axons, and the lengths after completion**.

	**Apical and basal dendrites (6552 μm)**				**Axons (10655 μm)**			
	**Length cut dendrites**		**Length completed dendrites**		**Length cut axons**		**Length completed axons**	
**Slice thickness**	**Length (μm)**	**Deviation (%)**	**Length (μm)**	**Deviation (%)**	**Length (μm)**	**Deviation (%)**	**Length (μm)**	**Deviation (%)**
**EXCLUDING ORPHAN BRANCHES**								
**Central soma positions in slice**								
100	4294	34.5	6065	7.4	1505	85.9	4148	61.1
200	5764	12.0	6446	1.6	4140	61.1	7926	25.6
300	6288	4.0	6553	0.0	6684	37.3	9817	7.9
400	6481	1.1	6557	−0.1	8225	22.8	10,356	2.8
500	6544	0.1	6551	0.0	9143	14.2	10,457	1.9
600	6551	0.0	6551	0.0	9791	8.1	10,615	0.4
1000	6552	0.0	6552	0.0	10,567	0.8	10,655	0.0
2000	6552	0.0	6552	0.0	10,655	0.0	10,655	0.0
**Random soma positions in 80% range of slice thickness (10–90%)**								
100	3856	41.1	5945	9.3	1484	86.1	4125	61.3
200	5264	19.7	6399	2.3	4239	60.2	8531	19.9
300	5818	11.2	6445	1.6	6428	39.7	10,350	2.9
**Random soma positions in 60% range of slice thickness (20–80%)**								
100	4082	37.7	6086	7.1	1581	85.2	4481	57.9
200	5513	15.9	6440	1.7	4109	61.4	7993	25.0
300	6069	7.4	6499	0.8	6594	38.1	10313	3.2
**INCLUDING ORPHAN BRANCHES**								
**Central soma positions in slice**								
100	4363	33.4	6257	4.5	2748	74.2	9907	7.0
200	5777	11.8	6468	1.3	5155	51.6	10,421	2.2
300	6288	4.0	6553	0.0	7055	33.8	10,568	0.8
400	6481	1.1	6557	−0.1	8362	21.5	10,560	0.9
500	6544	0.1	6551	0.0	9213	13.5	10,562	0.9
600	6551	0.0	6551	0.0	9813	7.9	10,647	0.1
1000	6552	0.0	6552	0.0	10,567	0.8	10,655	0.0
**Random soma positions in 80% range of slice thickness (10–90%)**								
100	4052	38.2	6352	3.1	2817	73.6	10,285	3.5
200	5316	18.9	6475	1.2	5192	51.3	10,933	−2.6
300	5842	10.8	6478	1.1	6875	35.5	11,165	−4.8
**Random soma positions in 60% range of slice thickness (20–80%)**								
100	4196	36.0	6325	3.5	2799	73.7	10,164	4.6
200	5534	15.5	6472	1.2	5199	51.2	10,734	−0.7
300	6076	7.3	6508	0.7	7019	34.1	11,052	−3.7

Results are shown for 50 sliced NETMORPH neurons for several slice thicknesses, with central or random positions of somata within the slices, and excluding or including orphan branches. The original neurons had a mean dendritic length of 6552 μm and a mean axonal length of 10655 μm.

Table 1 summarizes the effects of slicing on the dendrites and axons of 50 NETMORPH-generated neurons, depending on slice thickness and soma positions in the slice. Also are shown the results of the completion algorithm, taking or not taking into account the orphan branches. The original mean (apical and basal) dendritic and axonal lengths are 6552 and 10655 μm, respectively.

As expected, slicing has a significant effect on the length of axons and dendrites. For instance, centrally located neurons in 100 μm thick slices loose 34.5% of their dendritic length and 85.9% of their axonal length; in 200 μm thick slices the neurons loose 12.0% of their dendritic length and 61.1% of their axonal length; and in 300 μm thick slices they loose 4.0% of their dendritic length and 37.3% of their axonal length. A significant recovery of the lost parts was obtained by applying the completion procedure. For instance, when orphan branches were not included, dendritic loss in 100 μm thick slices was reduced from 34.5% down to 7.4%, and axonal loss from 85.9 to 61.1%. When orphan branches were included, dendritic loss was further reduced to 4.5% and axonal loss to 7.0%. In 300 μm thick slices, dendritic recovery of centrally located neurons excluding orphan branches resulted in 0% dendritic loss and 7.9% axonal loss, and including orphan branches in 0% dendritic loss and 0.8% axonal loss.

When the neurons were randomly placed in the slice, the losses were slightly higher for the dendrites, but more or less similar for the axons because the larger extent of the axons makes them less sensitive to the precise position in the slice. Also with random placement the completion procedure was able to greatly reduce the loss. For instance, in the case of a Z-range of 10–90%, the dendritic loss of 41.1% in 100 μm thick slices was reduced by completion without orphan branches to 9.3%, and with orphan branches to 3.1%. Axonal loss of 86.1% was reduced by completion without orphan branches to 61.3%, and with orphan branches to 3.5%.

The results in Table 1 show that the completion procedure that takes orphan branches into account is able to fully recover the original mass, with loss values around zero. Small negative loss values also occur, indicating an overcompensation, which can be expected when, as a result of statistical fluctuations in the spatial distribution of the arbors, the mass densities inside the slice are somewhat larger than outside the slice. The full recovery of dendritic and axonal mass when orphan branches are included can be considered as a validation of the completion procedure. In the absence of knowledge about orphan branches, which is usually the case, the completion procedure is still able to recover dendritic mass to values very close to the original mass, with a deviation of less than 2.3% in the case of 200 μm thick slices, and less than 1.6% in 300 μm thick slices.

Intracortical axons extend their branches at large distances from the slice and their loss in 100 μm and 200 μm thick slices is substantial (up to 85.9 and 61.1%, respectively). Although the completion procedure significantly reduces these losses (to 61.1% in 100 μm thick slices, to 25.6% in 200 μm thick slices, and to 7.9% in 300 μm thick slices), knowledge of orphan branches is required for a full recovery.

The loss of dendritic and axonal mass by slicing depends on the spatial extent of dendritic and axonal arbors in relation to the slice thickness. The NETMORPH neurons have a mean radial center of dendritic mass at 70 μm, and a mean radial center of axonal mass at 204 μm. The dependence of mass loss on slice thickness as well as the results from the completion procedure is depicted in Figure 5, displaying the mass loss of neurons with their somata in the center of the slice. Clearly is shown that for 300 μm thick slices, completion without orphan branches results in a full recovery of the dendrites (dashed red curve in 5A), with a loss of 0% (Table 1), and an almost full recovery of the axons (dashed green curve in 5B), with a loss of 7.9% (Table 1).

Figure 5C shows a scatter plot of the mass losses of the axonal and dendritic arborizations of each individual neuron vs. the radial center-of-mass of these arborizations after slicing. These data were obtained for neurons with their somata uniform randomly positioned in a 80% range of the slice thickness of 300 μm. Thus the closest distance of a soma to the cutting plane is 30 μm. Figure 5D shows a scatter plot of the recovery results vs. the radial center-of-mass of the individual axons and dendrites within the slice. Figures 5C,D reveal a large scatter in the individual data points, originating from the large variability in morphologies of the axonal and dendritic arborizations and the effect of cutting. The larger extent of axonal arborizations in comparison with dendritic arborizations (as reflected by the larger values for their radial center-of-masses) causes also larger values for their mass losses by slicing, as shown in Figure 5C. Nevertheless, for these 300 μm thick slices, there is almost full recovery of both axons (95.7%) and dendrites (98.3%). Note that the axonal recovery is better than shown in Figure 5A because of the different positions of the somata in the slices. The uniform random positions of somata are more realistic than a central positioning. The findings in Figure 5 thus show that the completion procedure results in (almost) full recovery of the mass distributions of axonal and dendritic arborizations when they were cut by 300 μm thick slices. The amount of scatter in the data points appears to depend also on the locations of the somata in the slices. In Supplementary Figure S5, the results are also shown for centrally located neurons, and for uniform random placements in a 60% range of the 300 μm thick slices.

Whether recovery results are related to the fraction of the original neuronal mass conserved in the slice is shown in Figure 5E using the data in Table 1 (excluding orphan branches). Both the axonal (green) and dendritic (red) data points show a clear dependency of recovery result on the fraction of conserved mass. The data points are labeled by the section thickness and the positioning scheme used. Averaged over the three positioning schemes we observe for the dendrites, that a conserved mass of 62.2% (100 μm) relates to a recovery of 91.8%, a conserved mass of 84.1% (200 μm) relates to a recovery of 98.1%, and a conserved mass of 92.5% (300 μm) relates to a recovery of 99.2%. For the axons, a conserved mass of 14.3% (in 100 μm thick slices) relates to a recovery of 39.9%, a conserved mass of 39.1% (200 μm) relates to a recovery of 76.4%, and a conserved mass of 61.6% (300 μm) relates to a recovery of 95.3%. Clearly is shown in this figure how the red dendritic data points for 100–300 μm thick slices intermingle with the green axonal data points for 300–1000 μm thick slices. Thus dendrites and axons show similar relationships obtained for different slice thicknesses, which leads us to the conclusion that recovery results relate to the fraction of conserved mass, while this relation is independent of the slice thickness.

### Data svoboda

**Figure 6:**
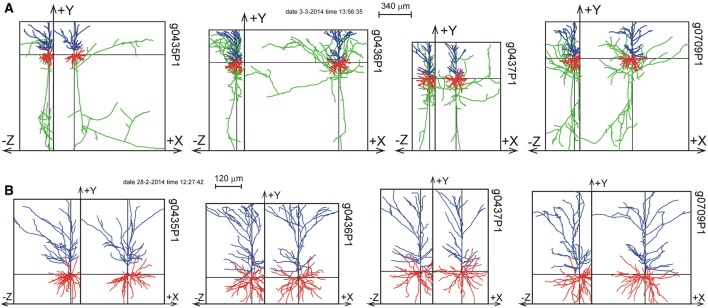
**Selection of 4 out of 11 reconstructions of rat cortical L2/3 pyramidal neurons (Shepherd and Svoboda, 2005) plotted as projections on the XY and YZ planes**. The neurons are aligned with their apical main stem into the Y-axis. **(A)** Plot of axons and dendrites with axons in green, basal dendrites in red and apical dendrites in blue. **(B)** Plot of apical and basal dendrites only. A full display of all the 11 reconstructions is given in Supplementary Figure S1.

The 11 rat parietal cortical L2/3 pyramidal neurons were reconstructed from 300 μm thick slices (Shepherd and Svoboda, 2005). Figure 6 shows a selection of four of these neurons as projections on the XY plane and the YZ plane. A full display of all 11 neurons is shown in Supplementary Figure S1. While the XY projections show the full extent of the arbors, the YZ projections clearly show the accumulation of terminal tips at the intersection of the XY and YZ planes.

**Figure 7:**
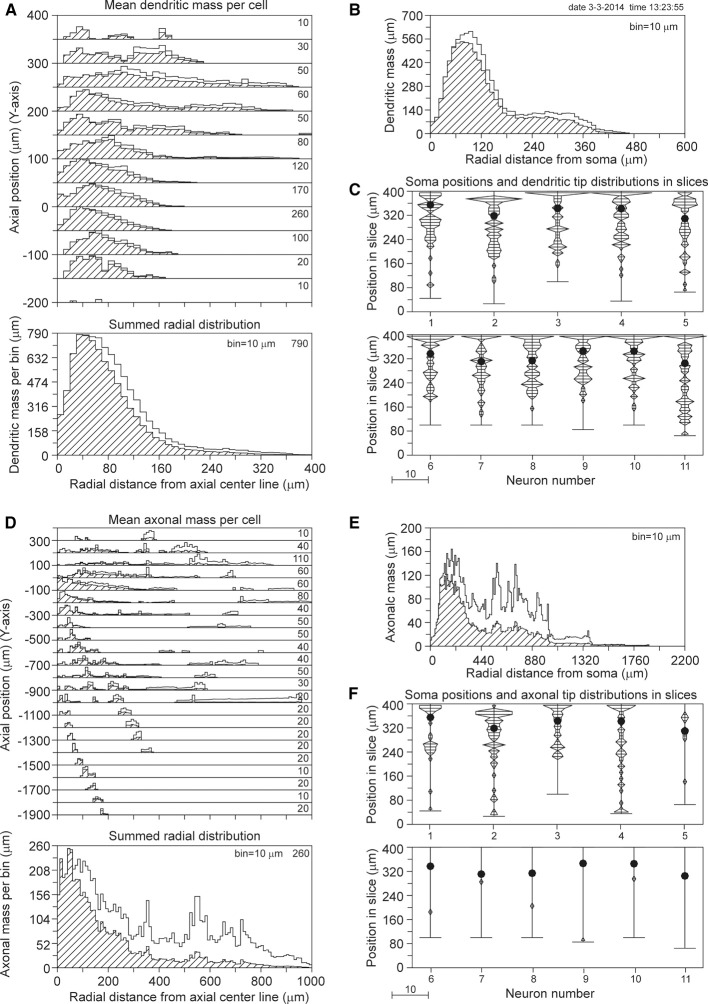
**Mass completion of rat cortical L2/3 pyramidal neurons reconstructed from 300 μm thick slices by Shepherd and Svoboda (2005), and obtained from the NeuroMorpho.org database**. For the description of the different panels see the legend of Figure 4. Lacking information on the soma positions in the slices, we aligned the neurons at the side with an accumulation of cut endings as shown in panels **(C)** and **(F)**. Because for some neurons the Z-range was larger than 300 μm, the Z-range in the panel was set at 400 μm with the neurons aligned at their cut endings at the top of the panel.

The axial-radial mass distributions of the reconstructed neurons after mass completion are shown in Figures 7A,D for (apical and basal) dendrites and axons, respectively. Particularly the summed radial distributions in Figures 7A,D and the radial to soma distributions in Figures 7B,E show the extent of the corrections, which were much larger for the axons than for the dendrites. Figures 7C,F clearly show the cut endings, which appear as an accumulation of cut terminal tips at one side of the arborizations. This accumulation was used as a criterion to align the neurons within their slices. The total length of the completed mass distributions is shown in Table 2A. The mean dendritic length increased from 7865 to 9289 μm, and the mean axonal length from 4692 to 9118 μm. The radial center-of-mass of the reconstructed dendrites was equal to 76 μm and that of the reconstructed axons 195 μm. Based on the validation results with the NETMORPH neurons (Figure 5) and the thickness of the slices in the Svoboda data set (300 μm), we may expect an almost full recovery of the axonal and dendritic mass. This implies that by slicing 15% of the dendritic mass was lost, and 49% of the intracortical axonal mass (within an uncertainty range of a few percent).

**Table 2:** **Outcomes of the completion procedure applied to (A) the dataset of Svoboda, (B) the dataset of Markram, and (C) the Parnavelas-Uylings datasets of all age groups in the developmental study, viz. 10, 14, 18, 24, 30, and 90 days PN**.

**A. COMPLETION RESULTS OF SVOBODA RECONSTRUCTIONS**						
Slice thickness 300 μm	**Length apical and basal dendrites (μm)**			**Length axons (μm)**		
	**Actual**	**Completed**	**Mass loss (%)**	**Actual**	**Completed**	**Mass loss (%)**
	7865	9289	15	4692	9118	49
**B. COMPLETION RESULTS OF MARKRAM RECONSTRUCTIONS**						
Slice thickness 300 μm	**Length apical and basal dendrites (μm)**			**Length axons (μm)**		
	**Actual**	**Completed**	**Mass loss (%)**	**Actual**	**Completed**	**Mass loss (%)**
	3790	4567	17	3211	6177	48
**C. COMPLETION OF PARNAVELAS-UYLINGS RECONSTRUCTIONS**						
Slice thickness 120 μm	**Length basal dendrites (μm)**					
**Age groups (PN) (No.)**	**Actual**	**Completed**	**Mass loss (%)**			
10 (30)	419	446	6			
14 (28)	826	935	12			
18 (24)	1368	1568	13			
24 (24)	938	1036	9			
30 (23)	1041	1160	10			
90 (24)	2022	2532	20			

The data represents the actual measured axonal and dendritic length from the reconstructions, the recovered length after the completion procedure, and an estimate of the mass loss (%). Note that the mass loss (%) is estimated relative to the recovered length (and not relative to the true length, which is unknown).

### Data markram

**Figure 8:**
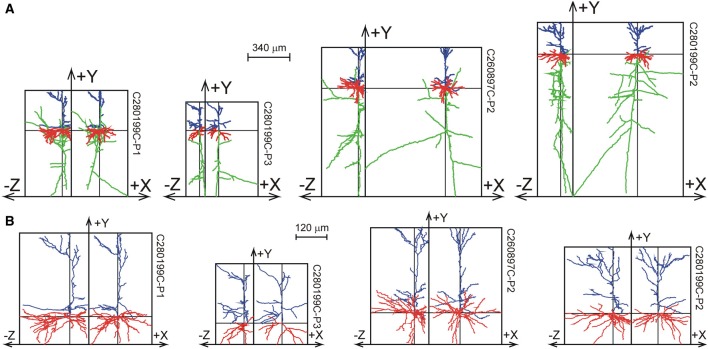
**Selection of reconstructions of 33 Wistar rat cortical L2/3 pyramidal neurons (Wang et al., 2002) plotted as projections on the XY and YZ planes. (A)** Projections of both axons and dendrites with axons in green, basal dendrites in red and apical dendrites in blue. The basal and apical dendrites are separately shown in **(B)**. The neurons are aligned with their apical main stem into the Y-axis. A full display of the 33 neurons is given in Supplementary Figures S2, S3.

The 33 Wistar rat somatosensory cortical L2/3 pyramidal neurons were reconstructed by Wang et al. (2002) from 300 μm thick slices, and are shown in Figure 8 as projections on the XY plane and the YZ plane. While the XY projections show the full extent of the arbors, the YZ projections clearly show the accumulation of terminal tips at the intersection of the XY and YZ planes.

**Figure 9:**
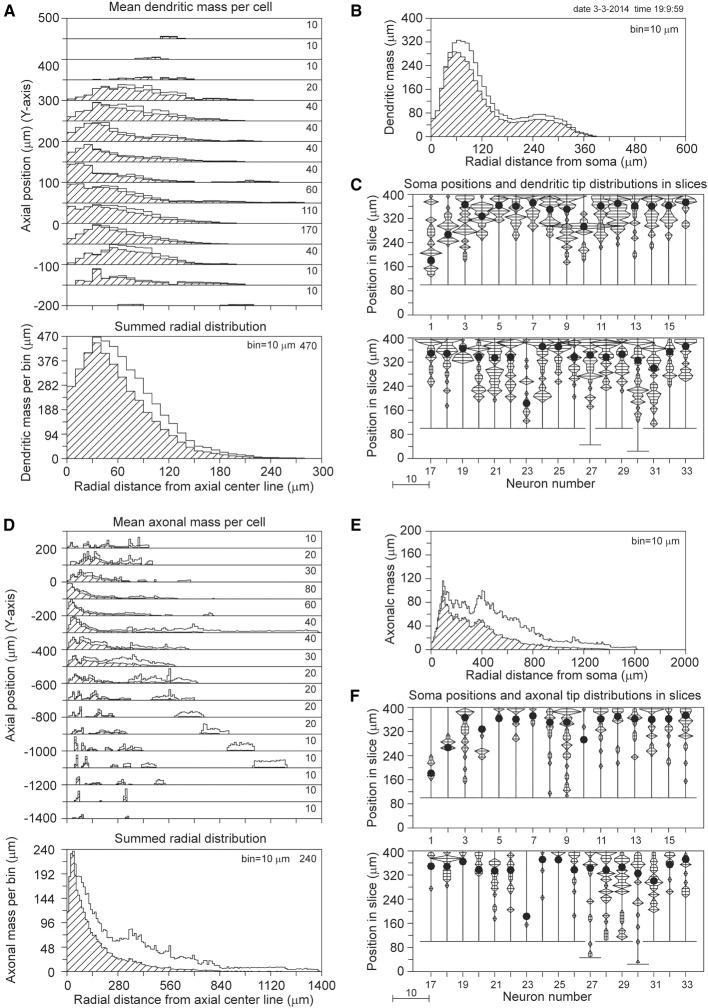
**Mass completion of 33 Wistar rat cortical L2/3 pyramidal neurons reconstructed from 300 μm thick slices by Wang et al. (2002), and obtained from the NeuroMorpho.org database**. For the description of the different panels see the legend of Figure 4. Lacking information on the soma positions in the slices, we aligned the neurons at the side with an accumulation of cut endings as shown in panels **(C)** and **(F)**. Because for two neurons the Z-range was larger than 300 μm, the Z-range in the panel was set at 400 μm with the neurons aligned at their cut endings at the top of the panel.

The axial-radial mass distributions of the original neurons and after mass completion are shown in Figures 9A,D for (apical and basal) dendrites and axons, respectively. Particularly the summed radial distributions in Figures 9A,D and the radial to soma distributions in Figures 9B,E show the extent of the corrections, which were much larger for the axons than for the dendrites. Figures 9C,F clearly show the cut endings as an accumulation of cut terminal tips at one side of the arborizations, which was used as a criterion to align the neurons within their slices. The length of the completed mass distributions is shown in Table 2B. The mean dendritic length increased from 3790 to 4567 μm, and the mean axonal length from 3211 to 6177 μm. The radial center-of-mass of the reconstructed dendrites was 59 μm and that of the reconstructed axons 160 μm. Based on the validation results with the NETMORPH neurons (Figure 5) and the thickness of the slices in the Markram data set (300 μm), we may expect an almost full recovery of the axonal and dendritic mass. This implies that by slicing 17% of the dendritic mass was lost, and 48% of the intracortical axonal mass (within an uncertainty range of a few percent).

### Data parnavelas-uylings

**Figure 10:**
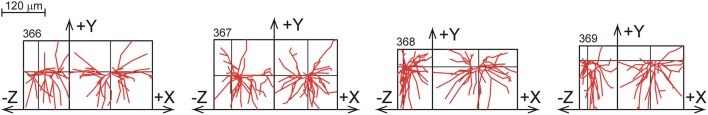
**A selection of rat visual cortex layer 2/3 pyramidal basal dendrites at the age of 90 days PN, reconstructed by Parnavelas-Uylings (Uylings et al., 1994), and plotted as projections onto the XY and YZ plane**. A full display of all the neurons is given in Supplementary Figure S4.

The data from Parnavelas and Uylings originate from a study of (basal) dendritic development in rat visual cortex pyramidal neurons (Uylings et al., 1994). Golgi stained dendrites were reconstructed from slices with a thickness of about 120 μm. Reconstructions were obtained from 153 layer 2/3 pyramidal cells at different ages of cortical development, i.e., at 10, 14, 18, 24, 30, and 90 days postnatal (PN). Figure 10 shows a selection of the 24 L2/3 pyramidal reconstructions at the age of 90 days PN. A full display of all the neurons is given Supplementary Figure S4.

**Figure 11:**
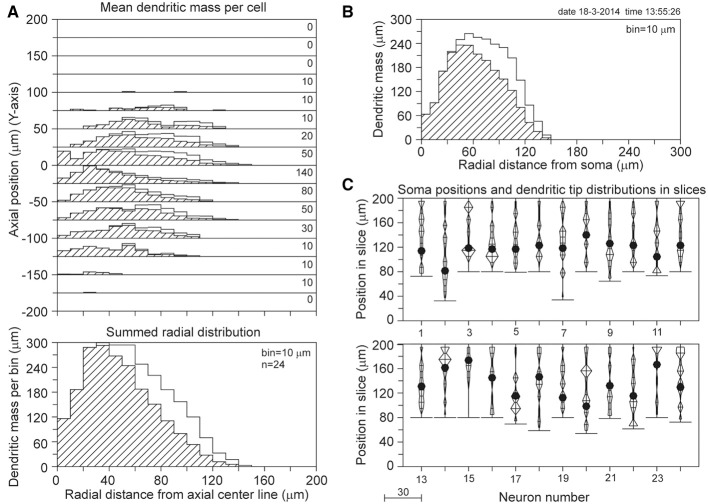
**Mass completion of basal dendrites of 24 rat visual cortex L2/3 pyramidal neurons (age 90 days PN), reconstructed from 120 μm thick slices by Parnavelas-Uylings (Uylings et al., 1994)**. For the description of the different panels see the legend of Figure 4. Lacking information on the soma positions in the slices, we aligned the neurons at the side with an accumulation of cut endings as shown in **(C)**. Because for some neurons the Z-range was larger than 120 μm, the Z-range in the panel was set at 200 μm with the neurons aligned at their cut endings at the top of the panel.

The results of the completion procedure for the 90 days PN data set are shown in Figure 11. With a mean length of 2022 μm for the reconstructed basal dendrites and 2532 μm for the completed masses, the mass loss by slicing becomes 20%. However, the validation study (Table 1) has shown that for 100 and 200 μm thick slices dendritic recovery still leaves a deficit of 7.1 and 1.7%, respectively, for somata within a 60% range of the slice thickness. Based on these validation findings, we may expect that the outcome of the completion procedure of 2532 μm deviates less than 7% from the original mean dendritic mass, thus in the range of 2532–2723 μm. The outcomes of the completion procedure for the other age groups are listed in Table 2C.

## Discussion

The aim of this study was to explore the feasibility of density field completion of incompletely reconstructed neurons. The underlying idea was that when the density of the arborizations within the slices can be estimated, an extrapolation to the space outside the slice is possible under the assumption of axial symmetry. The method for this extrapolation is based on simple geometrical considerations.

### Other approach for recovering cut arborizations

The problem of incomplete reconstructions by slicing has recently also been studied by Hill et al. (2012). The algorithm they devised for morphological repair was to derive a statistical growth model from the intact parts of the arborizations and then to regrow the cut portions. Using Bayesian spatial distributions, cut dendrites were regrown point by point. Axons were separately repaired by pasting subtrees from the intact parts. Invariance to axial rotations was also assumed. While Hill et al. (2012) attempted to recover the individual branches outside the slice volume, our approach aimed at recovering the population mean axonal and dendritic mass density fields by extrapolating the observed mass distributions within the slices to the outside space by simple geometrical relationships. Our approach is not model-based, and the only assumption used is that of axial symmetry in the mass distributions.

### Validation of the completion method

For the validation of the completion procedure we used a set of 50 neurons generated with our NETMORPH simulator. The neurons were subsequently sliced with different slice thicknesses, and subjected to the completion procedure. Comparison of the sliced masses and completed masses then showed how well the completion procedure was able to recover the lost masses. It turned out that a complete recovery was indeed possible provided that all the original mass of the sliced neurons within the slices was used. This outcome validated the completion procedure. However, the original mass within a slice includes the contiguous part seen from the soma, as well as the mass of orphan branches that lost their connection with the contiguous part within the slice. Because the orphan branches are not included in experimental reconstructions, they remain a missing part of the reconstructed neurons and therefore affect the degree of mass recovery. However, the number of orphan branches depends on the slice thickness and the spatial extent of the arborizations. From the validation data it turned out that this missing part for dendrites in the case of 300 μm thick slices was negligibly small, while for intracortical axons the missing part was less than 8% for centrally located neurons (Figure 5). For randomly located neurons in 300 μm thick slices a full (i.e., better than 98%) dendritic recovery and an almost full axonal recovery (i.e., better than 95%) was obtained by the completion procedure.

### Dependence on soma locations in slices

The validation study revealed that the mean loss of mass in a population of neurons depends on the positions of the somata within the slice. However, a systematic trend could not be derived from Table 1, because the outcomes for the three different location options also depended on the slice thickness and the extent of arborization (dendritic or axonal). Apparently, these geometrical parameters all play a role in the final outcome.

### Relation between conserved mass in slice and recovery result

Recovery results appear to relate to the fraction of conserved mass in the slice. Both the dendritic and the axonal data show a similar relationship. In particular the observation that dendritic data points in this relation for 100 μm thick slices coincide with axonal data points for 300 μm thick slices, and for 200 and 300 μm thick slices are intermingled with axonal data points for 400–1000 μm thick slices shows that this relationship is independent of the slice thickness itself. Thus, the fraction of conserved mass in the slice determines the level of recovery obtained. For instance, a recovery result better than about 95% requires a conserved mass in the slice larger than about 60%. This relation may be of practical value as it provides guidance for the required slice thickness which will be different for axons and dendrites. Whether a similar relationship between conserved mass and recovery result is obtained when the method is applied to experimentally fully reconstructed neurons instead of NETMORPH generated neurons is still an open question.

### Application to experimental data sets

Based on the positive outcomes of the validation study, we have analyzed three data sets of reconstructed neurons, two of which were obtained from the NeuroMorpho.org data base and one was provided by one of the authors of this study.

The Svoboda data set (Shepherd and Svoboda, 2005) resulted in a recovery of the mean dendritic length of 9289 μm (from the actual measured value of 7865 μm, indicating a loss by slicing of 15%). Axonal length was recovered up to 9118 μm (from the actual measured value of 4692 μm, indicating a mass loss of 49%), but this outcome may still be about 5% from the true value (see discussion in the previous two paragraphs).

The Markram data set (Wang et al., 2002) resulted in a recovery of the mean dendritic length of 4567 μm (from the actual measured value of 3790 μm, indicating a loss by slicing of 17%). Axonal length was recovered up to 6177 μm (from the actual measured value of 3211 μm, indicating a mass loss of 48%), but this outcome may still be about 5% from the true value (see discussion in the previous two paragraphs).

The Parnavelas-Uylings data set (Uylings et al., 1994) resulted in a recovery of the mean basal dendritic length of the 90 days age group of 2532 μm (from the actual measured value of 2022 μm, indicating a loss by slicing of 20%). These dendritic reconstructions were, however, made from 120 μm thick slices and the recovery result may still be about 7% from the true value (see Table 1, dendritic deviation for 100 μm thick slices with random positions in 20–80% range).

The outcomes of the three data sets are not directly comparable, because of different ages of the rats used (25–36 days PN for the Svoboda data, 13–15 days PN for the Markram data, and 90 days PN for the Parnavelas-Uylings data), and the restriction in the last data set to basal dendrites only.

It is interesting to note that the Svoboda and the Markram data set show similar mass losses for axons (48–49%) and dendrites (15–17%) in 300 μm thick slices. Apparently, the mean mass loss is not so sensitive to the difference in the mean radial center-of-masses of the cut axons (195 and 160 μm) and cut dendrites (76 and 59 μm) in the Svoboda and Markram data set, respectively. This is also in line with the more or less uncorrelated scatter of the individual NETMORPH neuron data in Figure 5C. Nevertheless, the axonal and dendritic losses do differ significantly in all these cases.

### Shrinkage of the tissue

The described completion/recovery method, and thus the quality of its outcomes, is independent of homogeneous tissue shrinkage. The 3D metrical properties of the arborizations, however, are affected by shrinkage. In quantifying the 3D geometry of neuronal arborizations, one needs to take tissue shrinkage into account, which occurs in histological and staining procedures. The extent of shrinkage is different for different staining techniques, and is even different for different Golgi techniques. For the Markram data set based on HRP staining, Wang et al. (2002) reported a 25% shrinkage of the slice thickness and ~10% anisotropic shrinkage along the X- and Y-axes. Only shrinkage of thickness was corrected. Shrinkage correction in the Svoboda data set (Shepherd and Svoboda, 2005) was not reported. No shrinkage correction was applied for the Parnavelas-Uylings data set (Uylings et al., 1994). Uylings et al. (1986, 1989) summarized tissue shrinkage values for different Golgi staining procedures. Linear shrinkage between 5 and 10% was reported for Golgi-Cox and rapid Golgi staining, and of 5–20% for Golgi-Kopsch staining. Thus, depending on histological and staining procedures, tissue shrinkage is an important issue in quantitative studies of 3D neuronal arborizations. Correction for shrinkage is only possible when the actual amount of anisotropic shrinkage in X, Y, and Z direction is known.

### Axial symmetry

A key assumption in the completion procedure is axial (rotational) symmetry in the distribution of axonal and dendritic mass. Because such symmetry is often assumed for pyramidal neurons, we have selected cortical layer 2/3 pyramidal neuron reconstructions for this study. While this assumption may be reasonably valid for basal dendritic arborizations that locally innervate space, it may not be valid for non-pyramidal dendrites (Parnavelas and Uylings, 1980) and axons that extend their arbors not only to local but also to remote locations. Clearly, single slice axonal reconstructions visualize only the local part of axonal arbors. Because the completion procedure recovers only this local axonal part, the axial symmetry assumption may still be valid.

### Density fields

The paper dealt with the estimation of the axial-radial distribution of axonal and dendritic mass. The calculation of axonal and dendritic densities is a straightforward extension and proceeds by dividing the mass in an integration ring (see Figure 2) by the volume of the ring (see Materials and Methods; see also Van Pelt and Van Ooyen, 2013).

### Density fields and synaptic connectivity estimation

Recently, we have shown that potential synaptic connectivity between neurons in a network can be estimated from their axonal and dendritic density fields (Van Pelt and Van Ooyen, 2013; McAssey et al., 2014). Thus, for constructing neuronal networks and their inter-neuron connectivity one does not need as many neuronal reconstructions as there are neurons in the network, but can use the population mean density fields instead. The availability of density fields for a variety of neuronal cell types is thus important. While for a large variety of cell types reconstructions have become available in open-access data bases, their incompleteness hampers a full use of the data. When with our method full density fields can be recovered from incomplete single-slice reconstructions, the open-access data become even more valuable, as they now can also be used for building neuronal networks and connectivity studies.

It has to be noted that the estimation of the number of synapses not only requires the number of potential synapse locations but also the probability that synapses actually are formed at these locations. A detailed EM study of the hippocampal neuropil by Mishchenko et al. (2010) showed that this probability was variable and dependent on ultrastructural details, such as dendritic circumference and actual axo-dendritic touches. Helmstaedter (2013) emphasizes in his review on dense neural circuit reconstruction that in “mapping neuronal circuits, it is important to detect synaptic contacts between neurons, but it is in many cases even more important to be able to exclude synaptic connectivity between neurons to determine the structure of a wiring diagram,” Clearly, the estimation of potential synapse locations is only one, but still crucial, factor in estimating synaptic connectivity.

Nevertheless, the realism of network connectivity estimates based on overlapping axonal and dendritic arborizations has recently been demonstrated by Hill et al. (2012) and Van Ooyen et al. (2014). In a statistical study, Hill et al. (2012) found that random alignment of axonal and dendritic arbors provides a sufficient foundation for specific functional connectivity to emerge in local neural microcircuits. In a computational study, Van Ooyen et al. (2014) found that the synaptic connectivity emerging between neurons that grow out in the absence of any guidance cues showed a good agreement with available experimental data on spatial locations of synapses on dendrites and axons, number of synapses by which neurons are connected, connection probability between neurons, distance between connected neurons, and pattern of synaptic connectivity.

### Conflict of interest statement

The authors declare that the research was conducted in the absence of any commercial or financial relationships that could be construed as a potential conflict of interest.

## Supplementary material

Supplementary Figures give a full display of the neurons obtained from the NeuroMorpho.org database and used for the application of the completion method. Additional scatter plots are given of the loss of mass by slicing and recovery by completion vs. the radial center-of-mass in the axonal and dendritic mass distribution of the sliced neurons.

## References

[B1] Ascoli G. A. (2006). Mobilizing the base of neuroscience data: the case of neuronal morphologies. Nat. Rev. Neurosci. 7, 318–324 10.1038/nrn1885 16552417

[B2] Helmstaedter M. (2013). Cellular-resolution connectomics: challenges of dense neural circuit reconstruction. Nat. Methods 10, 501–507 10.1038/nmeth.2476 23722209

[B3] Hill S. L. Wang Y. Riachia I. Schürmann F. Markram H. (2012). Statistical connectivity provides a sufficient foundation for specific functional connectivity in eocortical neural microcircuits. Proc. Natl. Acad. Sci. U.S.A. 109, E2885–E2894 10.1073/pnas.1202128109 22991468 PMC3479474

[B4] Koene R. A. Tijms B. Van Hees P. Postma F. De Ridder S. Ramakers G. (2009). NETMORPH: a framework for the stochastic generation of large scale neuronal networks with realistic neuron morphologies. Neuroinformatics 7, 195–210 10.1007/s12021-009-9052-3 19672726

[B5] McAssey M. P. Bijma F. Tarigan B. Van Pelt J. Van Ooyen A. De Gunst M. (2014). A morpho-density approach to estimating neural connectivity. PLoS ONE 9:e86526 10.1371/journal.pone.0086526 24489738 PMC3906031

[B6] Mishchenko Y. Hu T. Spacek J. Mendenhall J. Harris K. M. Chklovskii D. B. (2010). Ultrastructural analysis of hippocampal neuropil from the connectomics perspective. Neuron 67, 1009–1020 10.1016/j.neuron.2010.08.014 20869597 PMC3215280

[B7] Parnavelas J. G. Uylings H. B. M. (1980). The growth of non-pyramidal neurons in the visual cortex of the rat: a morphometric study. Brain Res. 193, 373–382 10.1016/0006-8993(80)90171-7 7388598

[B8] Peters A. (1979). Thalamic input to the cerebral cortex. Trends Neurosci. 2, 1183–1185 10.1016/0166-2236(79)90074-2

[B9] Shepherd G. M. Healy M. D. Singer M. S. Peterson B. E. Mirsky J. S. Wright L. (1997). Senselab: a project in multidisciplinary, multilevel sensory integration, in Neuroinformatics: An Overview of the Human Brain Project, ed Huerta S. H. K. M. F. (Mahwah, NJ: Lawrence Erlbaum Associates, Inc), 21–56

[B10] Shepherd G. M. G. Svoboda K. (2005). Laminar and columnar organization of ascending excitatory projections to layer 2/3 pyramidal neurons in rat barrel cortex. J. Neurosci. 25, 5670–5679 10.1523/JNEUROSCI.1173-05.2005 15958733 PMC6724876

[B11] Uylings H. B. M. Van Eden C. G. Hofman M. A. (1986). Morphometry of size/volume variables and comparison of their bivariate relations in the nervous system under different conditions. J. Neurosci. Methods 18, 19–37 10.1016/0165-0270(86)90111-1 3540468

[B12] Uylings H. B. M. Van Pelt J. (2002). Measures for quantifying dendritic arborizations. Netw. Comput. Neural Syst. 13, 397–414 10.1088/0954-898X/13/3/309 12222821

[B13] Uylings H. B. M. van Pelt J. Parnavelas J. G. Ruiz-Marcos A. (1994). Geometrical and topological characteristics in the dendritic development of cortical pyramidal and nonpyramidal neurons, in Progress in Brain Research, The Self-Organizing Brain: From Growth Cones to Functional Networks, Vol. 102, eds van Pelt J. Corner M. A. Uylings H. B. M. Lopes da Silva F. H. (Amsterdam: Elsevier), 109–123 10.1016/S0079-6123(08)60535-X 7800808

[B14] Uylings H. B. M. Van Pelt J. Verwer R. W. H. McConnell P. (1989). Statistical analysis of neuronal populations, in Computer Techniques in Neuroanatomy, ed Capowski J. J. (New York, NY: Plenum Press), 241–264

[B15] Van Ooyen A. Carnell A. De Ridder S. Tarigan B. Mansvelder H. Bijma F. (2014). Independently outgrowing neurons and geometry-based synapse formation produce networks with realistic synaptic connectivity. PLoS ONE 9:e85858 10.1371/journal.pone.0085858 24454938 PMC3894200

[B16] Van Pelt J. Carnell A. De Ridder S. Mansvelder H. D. Van Ooyen A. (2010). An algorithm for finding candidate synaptic sites in computer generated networks of neurons with realistic morphologies. Front. Comput. Neurosci. 4:148 10.3389/fncom.2010.00148 21160548 PMC3001749

[B17] Van Pelt J. Van Ooyen A. (2013). Estimating neuronal connectivity from axonal and dendritic density fields. Front. Comput. Neurosci. 7:160 10.3389/fncom.2013.00160 24324430 PMC3839411

[B18] Wang Y. Gupta A. Toledo-Rodriguez M. Wu C. Z. Markram H. (2002). Anatomical, physiological, molecular and circuit properties of nest basket cells in the developing somatosensory cortex. Cereb. Cortex 12, 395–410 10.1093/cercor/12.4.395 11884355

