# Supplementary material for: Axonal and dendritic density field estimation from incomplete single-slice neuronal reconstructions
Source: Front Neuroanat. 2014 Jun 25;8:54. doi: 10.3389/fnana.2014.00054 (PMC4069480; doi:10.3389/fnana.2014.00054)
Supplement: Supplementary file 1 [file DataSheet1.DOCX]

***SUPPLEMENTARY MATERIAL***

**Axonal and dendritic density field estimation from incomplete single-slice neuronal reconstructions**

***Jaap van Pelt^1*^, Arjen van Ooyen^1^, Harry B.M. Uylings^2^***

^1^ Computational Neuroscience Group, Department of Integrative Neurophysiology, Center for Neurogenomics and Cognitive Research (CNCR), VU University Amsterdam, The Netherlands

^2^ Dept. Anatomy & Neuroscience, VU University Medical Center, Amsterdam, The Netherlands

**^*^ Correspondence:**

Jaap van Pelt
Computational Neuroscience Group,
Department of Integrative Neurophysiology,

Center for Neurogenomics and Cognitive Research,

VU University Amsterdam,

De Boelelaan 1085, 1081 HV Amsterdam, the Netherlands

[j.van.pelt@vu.nl](mailto:jaap.van.pelt@cncr.vu.nl)


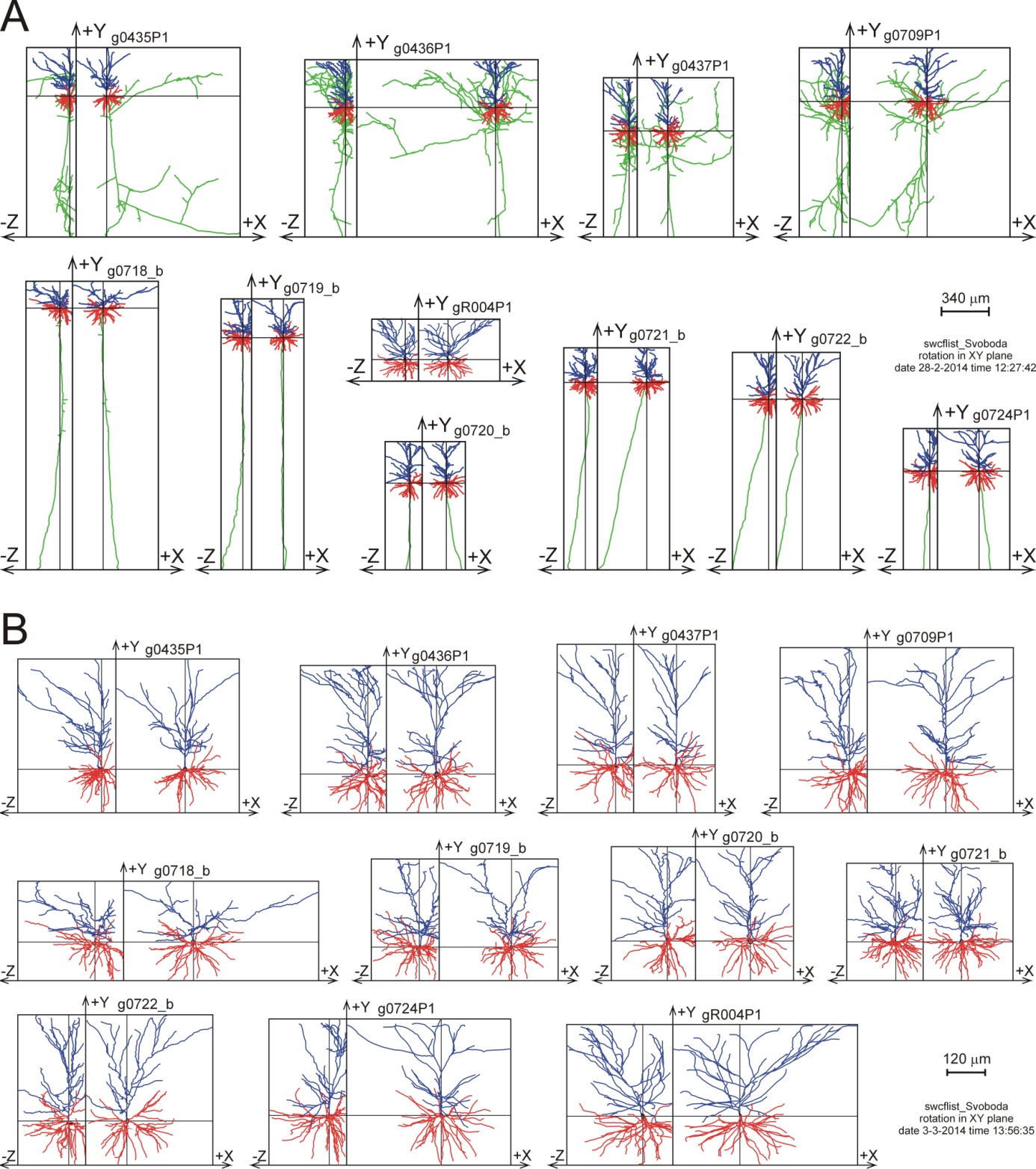


Supplementary Figure S1. Reconstructions of 11 rat cortical L2/3 pyramidal neurons (Shepherd and Svoboda, 2005) plotted as projections on the XY and YZ planes. The neurons are aligned with their apical main stem into the Y-axis. (A) Plot of axons and dendrites with axons in green, basal dendrites in red and apical dendrites in blue. (B) Plot of apical and basal dendrites only. Note that in (A) and (B) the neurons may appear in a different order.


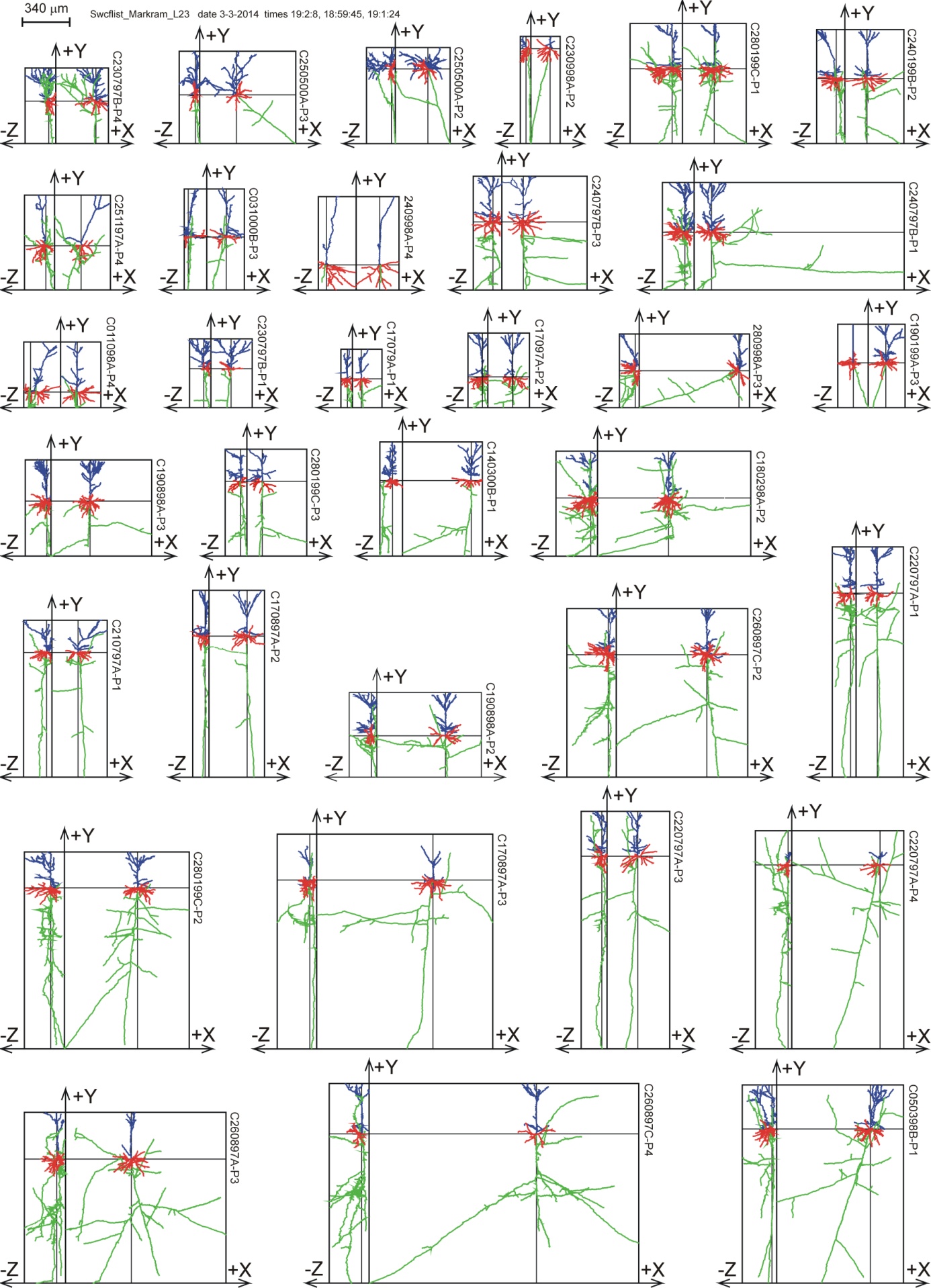


Supplementary Figure S2. Reconstructions of 33 Wistar rat cortical L2/3 pyramidal neurons (Wang et al., 2002) plotted as projections on the XY and YZ planes, with axons in green, basal dendrites in red and apical dendrites in blue. The neurons are aligned with their apical main stem into the Y-axis.


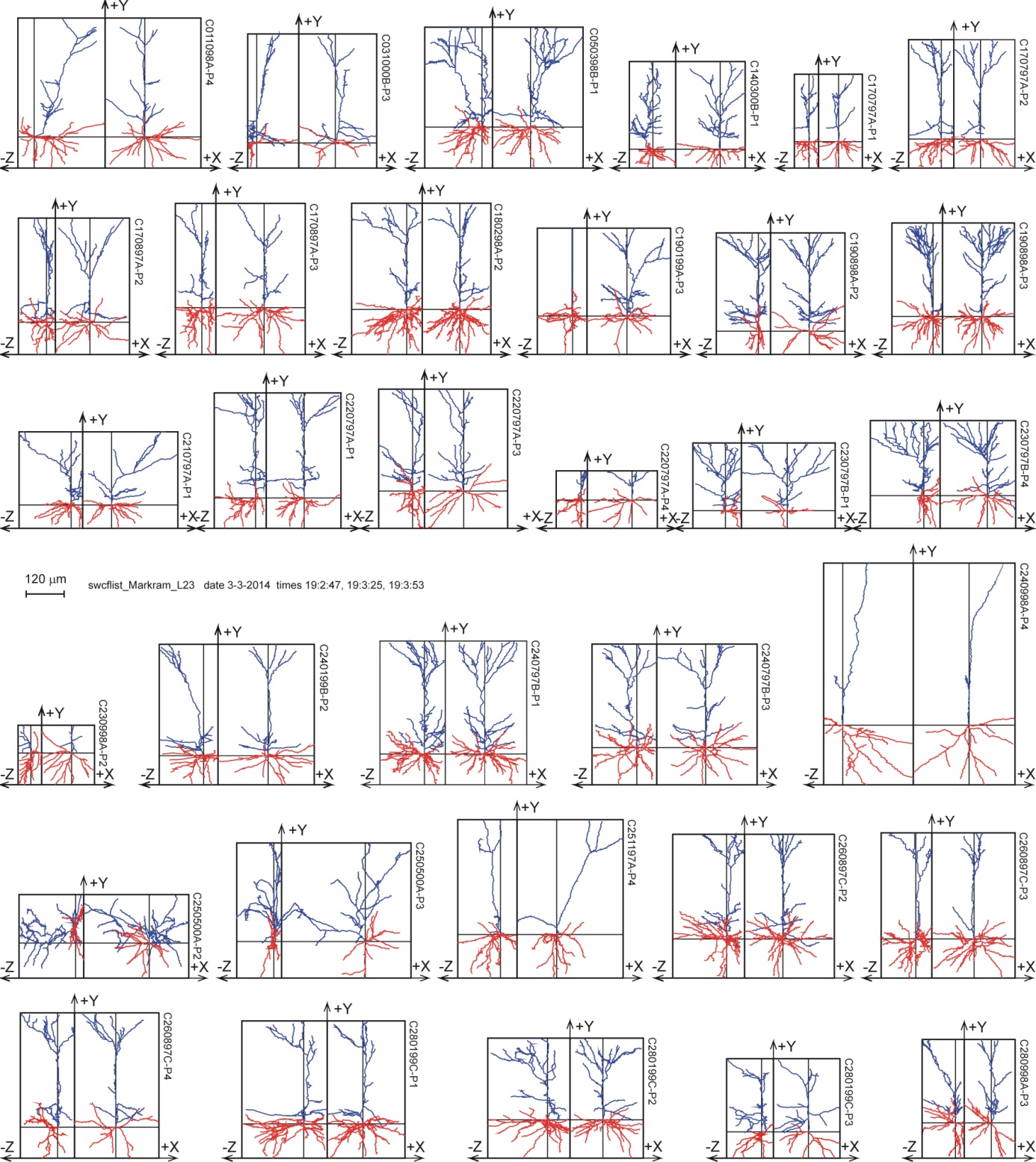


Supplementary Figure S3. Similar plot as Supplementary Figure S2 but restricted to basal and apical dendrites.


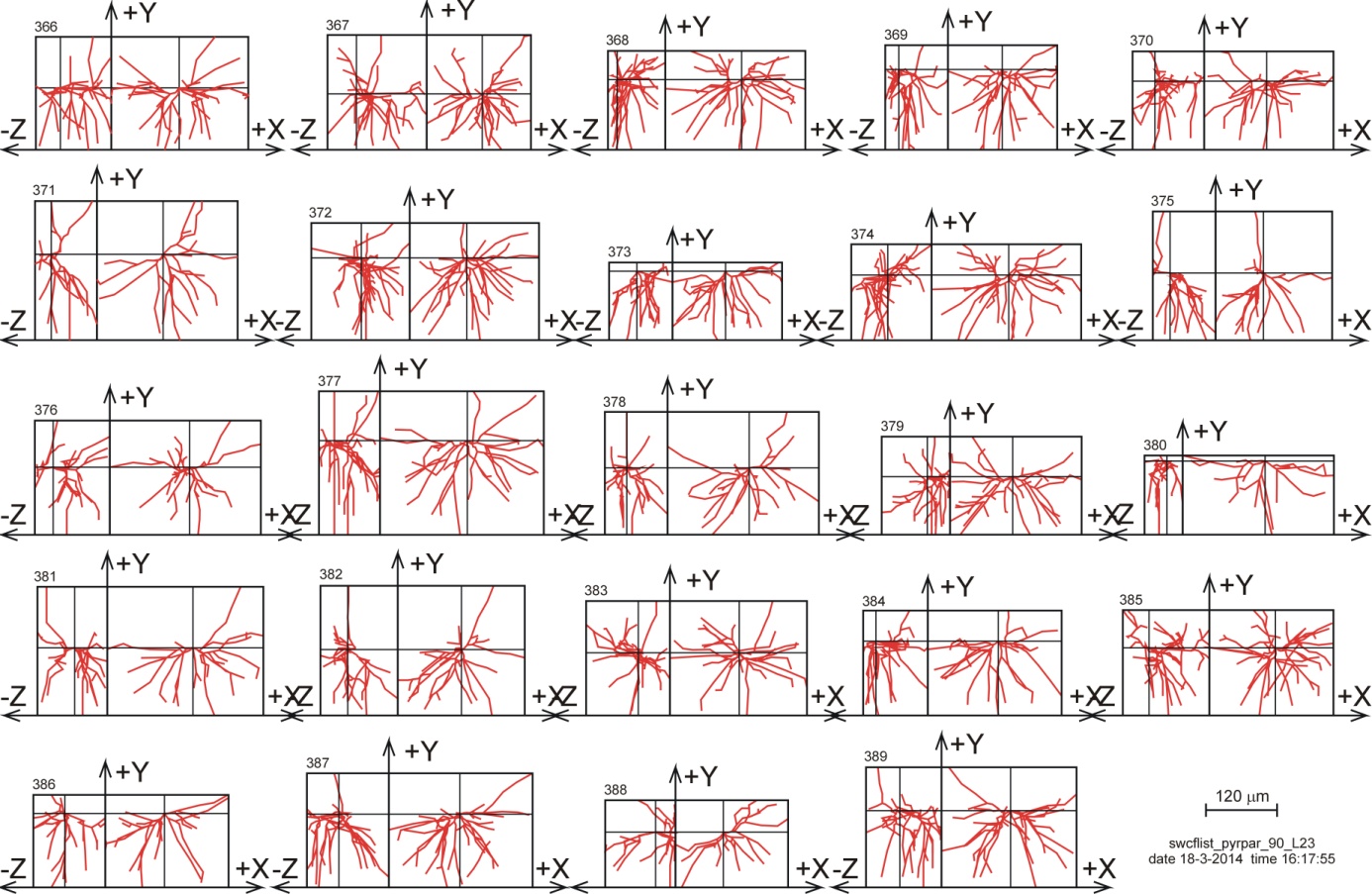


Supplementary Figure S4. Rat layer 2/3 visual cortex basal dendrites at the age of 90 days PN. Reconstructions from Panavelas-Uylings (Uylings et al., 1994), plotted as projections onto XY and YZ plane. The basal dendrites are aligned with the initial segment of the apical main stem into the Y-axis.


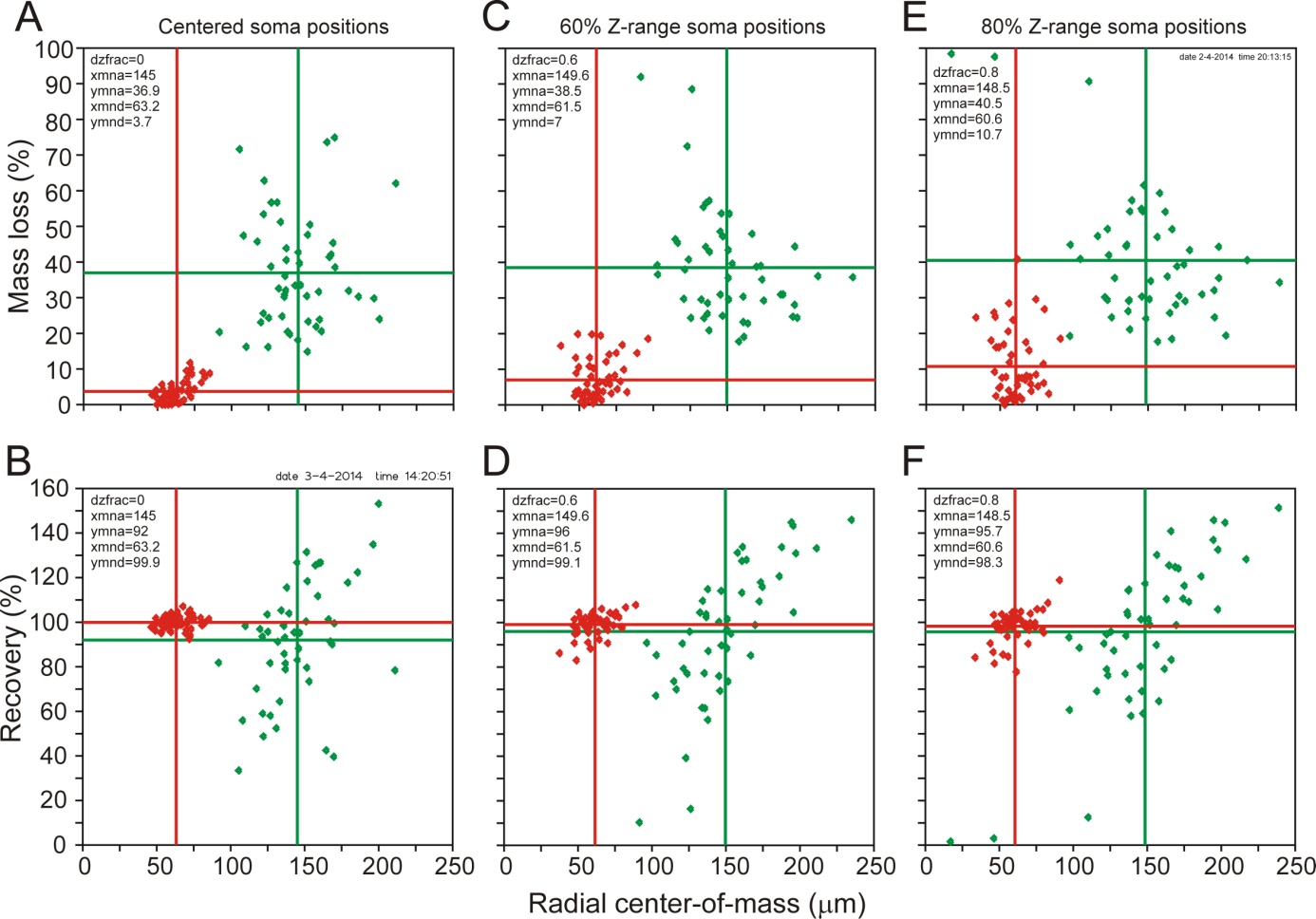


Supplementary Figure S5. Scatter plots of the loss of mass (top row) by sectioning and recovery by completion (excluding orphan branches) (bottom row) versus the radial center-of-mass in the axonal (green) and dendritic (red) mass distribution of the sliced neurons. The 50 NETMORPH-generated neurons are artificially sliced by 300 μm thick slices. The neuronal somata were (A,B) positioned in the center of the slice or uniform randomly positioned in (C,D) 60 % or (E,F) 80% of the slice thickness. The figures show the scatter of the individual data points in both the radial center-of-mass values, and in mass loss and recovery values, which is dependent on the position of the somata. The solid lines indicate the mean values for the point clouds, which correspond to the mean values in Table 1. Axons show a larger loss of mass by slicing than dendrites which is expected from their larger extents. The scatter within the individual point clouds originates from the variability in neuronal morphologies and their spatial distribution of mass. Thus also in the fraction of mass inside the slices, as result of which the completion procedure shows recoveries smaller or larger than 100%. The mean values for the axonal and dendritic recoveries for the whole population (point clouds), nevertheless, show values very close to 100%.
